# Concentration-Dependent Effects of Curcumin on Membrane Permeability and Structure

**DOI:** 10.1021/acsptsci.4c00093

**Published:** 2024-04-10

**Authors:** Jamie Gudyka, Jasmin Ceja-Vega, Katherine Ivanchenko, Zachary Morocho, Micaela Panella, Alondra Gamez Hernandez, Colleen Clarke, Escarlin Perez, Shakinah Silverberg, Sunghee Lee

**Affiliations:** Department of Chemistry and Biochemistry, Iona University, 715 North Avenue, New Rochelle, New York 10801, United States

**Keywords:** curcumin, 1,2-dioleoyl-*sn*-glycero-3-phosphocholine (DOPC), cholesterol, water permeability, differential scanning calorimetry, attenuated total reflectance-FTIR

## Abstract

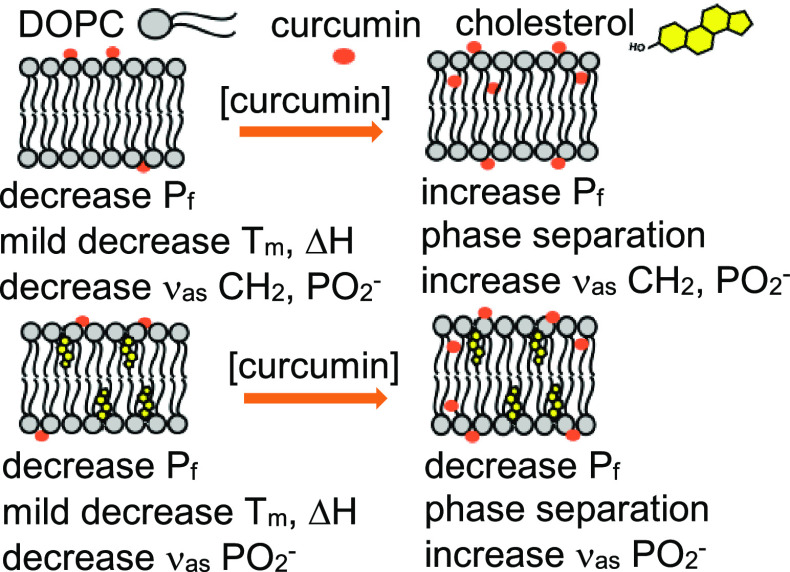

Growing evidence suggests that many bioactive molecules can nonspecifically modulate the physicochemical properties of membranes and influence the action of embedded membrane proteins. This study investigates the interactions of curcumin with protein-free model membranes consisting of 1,2-dioleoyl-*sn*-glycero-3-phosphocholine (DOPC) and DOPC with cholesterol (4/1 mol ratio). The focus is on the capability of curcumin to modify membrane barrier properties such as water permeability assayed through the droplet interface bilayer (DIB) model membrane. For pure DOPC, our findings show a concentration-dependent biphasic effect: a reduction in water permeability is observed at low concentrations (up to 2 mol %), whereas at high concentrations of curcumin, water permeability increases. In the presence of cholesterol, we observed an overall reduction in water permeability. A combination of complementary experimental methods, including phase transition parameters studied by differential scanning calorimetry (DSC) and structural properties measured by attenuated total reflectance (ATR)-FTIR, provides a deeper understanding of concentration-dependent interactions of curcumin with DOPC bilayers in the absence and presence of cholesterol. Our experimental findings align with a molecular mechanism of curcumin’s interaction with model membranes, wherein its effect is contingent on its concentration. At low concentrations, curcumin binds to the lipid–water interface through hydrogen bonding with the phosphate headgroup, thereby obstructing the transport of water molecules. Conversely, at high concentrations, curcumin permeates the acyl chain region, inducing packing disorders and demonstrating evidence of phase separation. Enhanced knowledge of the impact of curcumin on membranes, which, in turn, can affect protein function, is likely to be beneficial for the successful translation of curcumin into effective medicine.

Curcumin is a natural bioactive ingredient of the spice *Curcuma longa*, commonly known as turmeric. It has been used for centuries in both food and traditional medicine due to its perceived health and medicinal benefits.^1^ Curcumin, a polyphenolic phytochemical, is known to possess a wide range of beneficial pharmacological properties, including anti-inflammatory, antioxidant, and anticancer activities. It also holds potential promise as a therapeutic agent for neurodegenerative diseases such as Alzheimer’s and Parkinson’s.^2^ Consequently, a substantial body of scientific literature and human clinical trials have explored the use of curcumin-based therapies for the prevention and treatment of various diseases.^3−5^ Overall, understanding the biological impacts of curcumin has primarily focused on identifying particular pathways involving interactions with multiple enzymes, receptors, transporters, and signal transduction systems.^6,7^ However, the comprehensive molecular mechanism responsible for its pharmacological actions remains unclear.

Many studies have shown that curcumin affects a variety of molecular targets and signaling pathways^3,5^ and alters the function and expression of a broad range of structurally and functionally unrelated proteins. Yet its specific binding site on these target proteins has not been identified.^8^ Growing evidence suggests that many activities of phenolic phytochemicals including curcumin are associated with their ability to modulate the physicochemical properties of the membrane indirectly and nonspecifically, and thereby regulate the action of membrane proteins embedded in the host membrane.^9^ For instance, curcumin has been demonstrated to alter the function of membrane proteins, such as gramicidin A (gA), mechanosensitive channels of large conductance (MscL), K_V_2.1 channels, and various other membrane proteins, by affecting the physical properties of the lipid bilayer (ref (9) and references therein). In fact, curcumin is reported as one of the notorious pan-assay interference compounds (PAINS), which are molecules that yield false-positive assay results due to their nonspecific interactions with target proteins and their chemical modification.^4,10^ It is well recognized that lipid bilayers play a critical role in the physiological activities of transmembrane proteins, which constitute approximately 30% of the mammalian proteome and represent the primary category of targets for approved pharmaceutical drugs.^11,12^ These proteins sensitively respond to the surrounding lipid membrane matrix for their structural and functional integrity; therefore, perturbation of embedded proteins can result in significant changes in their biological functions, including maintenance of homeostasis, protein activity, and the propagation of cellular signaling processes.^13−16^

Despite curcumin’s demonstrated potential in treating various diseases, as evidenced by clinical and preclinical trials, its progression to becoming an FDA-approved drug has faced obstacles.^4,17^ Curcumin exhibits poor water solubility of 0.6 μg/mL,^18^ resulting in low bioavailability in the biological system. Additionally, due to its lipophilicity with a log P of approximately 3,^3^ its interactions with cellular membranes result in a low level of curcumin in the cytoplasm. To enhance the pharmacodynamics and accessibility of curcumin and other lipophilic plant-derived compounds, there is a growing utilization of nanoformulation-based techniques, including curcumin-loaded liposomes.^18,19^ Successful development of these drug-delivery vehicles necessitates a deeper comprehension of the membrane interaction with encapsulated compounds for engineering effective liposomal formulations of curcumin.^20^ Further investigations into the nature and extent of interactions involving curcumin and protein-free membranes are needed to better understand curcumin’s nonspecific effects, which in turn contribute to the overall effectiveness and biocompatibility of curcumin liposomal formulations.

Previous studies, both experimental and computational, have demonstrated the significance of nonspecific interactions between curcumin and lipid bilayer membranes. These interactions result in the modulation of collective membrane physical properties, including biomechanical, structural, electrical, and thermodynamic characteristics. These properties play a crucial role in physiological functions, such as cell fusion, cell division, endocytosis, and exocytosis. Nevertheless, there is no consistent agreement on the detailed molecular mechanism of action, including the location and orientation of curcumin on the lipid membrane.^21^ For example, curcumin has been proposed to reside in different locations within lipid bilayers, depending on its concentration^22^ and membrane hydration conditions as influenced by biological environments, such as buffers, substrates, or macromolecular solutes.^23^ These locations include: (1) at the interface between the lipid headgroup and hydrocarbon chain, with its long axis parallel to the plane of the membrane (membrane interface or carpet model), or (2) in the hydrophobic chain region of the bilayer, with its long axis perpendicular to the plane of the membrane (transbilayer or insertion model), or (3) a combination of (1) and (2).

Furthermore, it is still unclear whether curcumin induces disorder or order in the lipid bilayer. The addition of curcumin has been reported to influence the function of the gramicidin A (gA) channel by increasing its lifetime and appearance rates in 1,2-dioleoyl-*sn*-glycero-3-phosphocholine (DOPC) bilayers, by decreasing the bilayer stiffness.^8^ X-ray lamellar diffraction experiments demonstrate a nonlinear membrane thinning effect by curcumin, as well as weakening of the elastic moduli of DOPC bilayers.^24^ Curcumin has also been reported to disorder 1,2-dipalmitoyl-*sn*-glycero-3-phosphocholine (DPPC) membranes.^25^ Additionally, curcumin increases acyl chain fluctuations in a fully hydrated bilayer of 1,2-dimyristoyl-*sn*-glycero-3-phosphocholine (DMPC) according to X-ray diffraction and molecular dynamics (MD) computer simulations.^23^ MD studies also confirm a nonlinear thinning effect of curcumin on various lipid bilayers, inducing disorder and increasing lipid mobility.^26,27^ Neutron scattering techniques have shown curcumin to enhance the lateral motion of DPPC bilayers in both ordered and fluid phases.^21^ Furthermore, fluorescence spectroscopy has revealed that curcumin, at high concentrations, forms segregated domains that fluidize DMPC bilayer membranes in both solid gel and liquid crystalline phases.^22^ In contrast, some studies have reported that the lateral diffusion coefficient of lipids in DMPC and DOPC is reduced by the addition of curcumin, as studied by ^1^H NMR diffusometry.^28^ Electron paramagnetic resonance (EPR) spin labeling technique indicates that curcumin enhances membrane lipid order in DMPC and 1,2-distearoyl-*sn*-glycero-3-phosphocholine (DSPC).^29^ Solid-state NMR demonstrates curcumin’s strong effect on the dynamics of DMPC bilayers, altering hydrocarbon chain packing and increasing overall membrane order.^30^ Curcumin’s higher ordering effect is reported at lower concentrations, while at higher concentrations, there is a reduction in membrane thickness and molecular order.^30^

Because cellular biological membranes are exceptionally intricate,^31,32^ researchers have turned to model lipid membranes as a controlled and systematic means of gaining insight into the role of lipids in drug-membrane interactions. One such model membrane platform, known as the droplet interface bilayer (DIB), is established when lipid monolayers encase aqueous microdroplets and are brought into contact, resulting in the creation of a lipid bilayer region. This bilayer structure closely mimics the double-leaflet lipid bilayer found in cellular biological membranes.^33,34^ This interdroplet contact zone serves as a versatile platform for biomimetic modeling of cellular membranes and presents unique opportunities for investigating chemistry at the nanoscale within lipid bilayers. In our earlier studies, we established a DIB-based method to investigate water permeation barrier function in biomimetic membranes of diverse compositions.^35,36^ Water transport is crucial for cellular physiology and homeostasis maintenance. By using water as a molecular probe, the DIB offers a flexible platform to explore the influence of the lipid structure on passive permeation through bilayer aggregates. Our observations indicate that water transport across a single unsupported bilayer is highly responsive to the physical state of model membranes, revealing subtle compositional and structural effects,^37−40^ and selective influence of exogenous bioactive molecules.^41−44^

In this study, we investigated the impact of curcumin on the water transport properties across lipid membranes in the presence of varying curcumin concentrations, in the range of 100:1 to 4:1 (lipid to curcumin mol ratio). We employed a DIB platform composed of DOPC and cholesterol as model lipid membranes. By incorporating cholesterol into our model membrane system, we can more accurately replicate biological membranes and investigate how curcumin interacts with this binary system. In addition to determining water permeability, we utilized differential scanning calorimetry (DSC) to examine thermotropic properties and attenuated total reflection (ATR)-FTIR to analyze conformational changes, together providing complementary insights into the interaction of curcumin with DOPC and DOPC-cholesterol membranes. The structures of DOPC, cholesterol, and curcumin molecules are shown in Figure 1.

**Figure 1:**
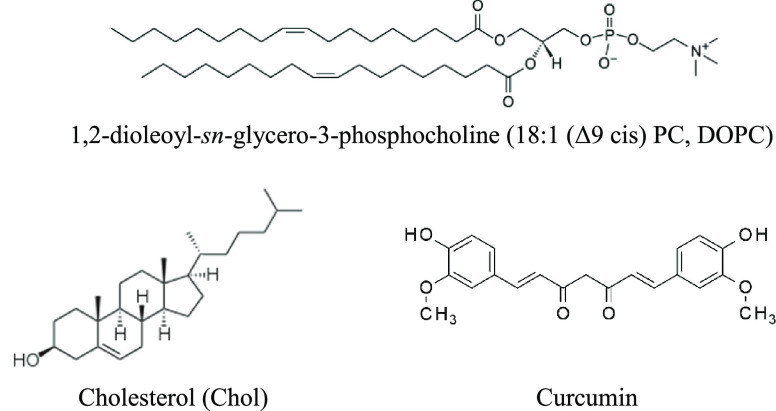
Structures of DOPC, cholesterol, and curcumin molecules.

## Results and Discussion

### Effect of Curcumin on Water Permeabilities of the DOPC and DOPC/Chol Membranes

**Figure 2:**
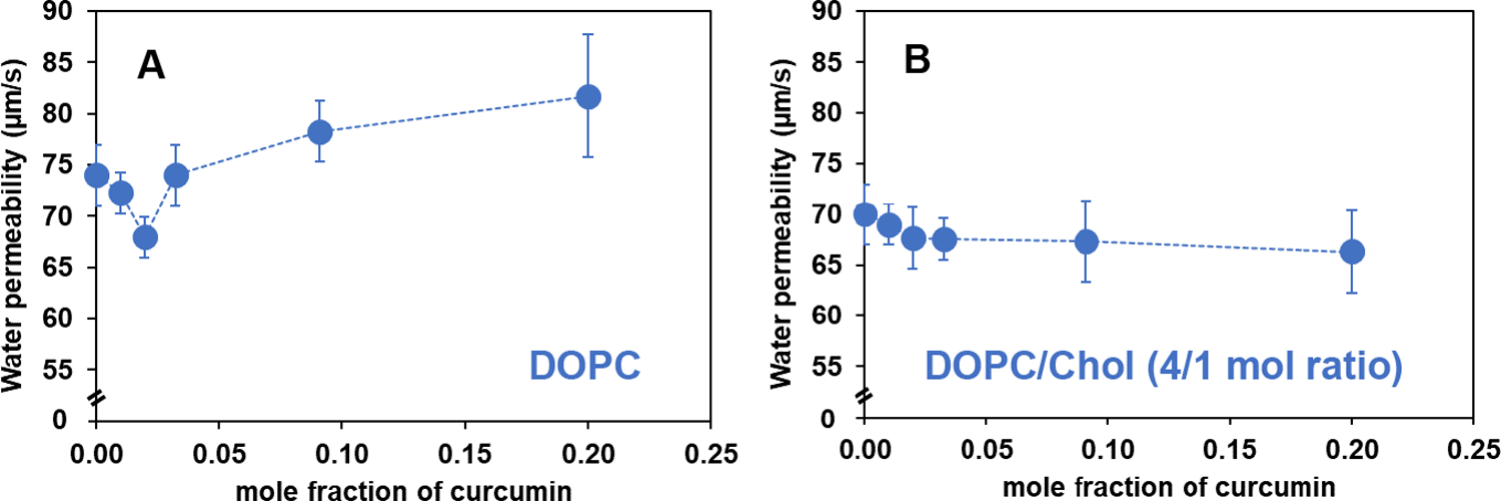
Osmotic water permeability coefficients (μm/s) of the lipid bilayer formed from (A) DOPC and (B) DOPC/Chol (4/1 mol ratio) at 30 °C with varying mole fractions of curcumin. Lines between data points in A and B are a guide to the eye.

The osmotic water permeability coefficients (*P*_f_) of model membranes composed of DOPC and DOPC/Chol at a 4/1 mol ratio at 30 °C are depicted in Figure 2 as a function of varying mole fractions of curcumin in the lipid mixture. The corresponding permeability coefficients are provided in Table S1 (Supporting Information). As shown in Figure 2A, our water permeability data for the DOPC membranes reveal a concentration-dependent effect of curcumin. At low concentrations of curcumin (100:1 and 50:1 DOPC to curcumin mole ratio), the *P*_f_ values for water transport across DOPC membranes decrease with increasing concentration of curcumin. At the lowest curcumin concentration employed (100:1 mol ratio of DOPC to curcumin), we observed a decrease in *P*_f_ from 74 to 72 μm/s. This decrease in *P*_f_ persists with increasing curcumin concentration, reaching 68 μm/s at a 50:1 mol ratio of DOPC to curcumin, an ∼8% decrease in comparison to the *P*_f_ of the control (pure DOPC). However, at higher concentrations of curcumin, beginning at and above a 30:1 lipid:curcumin mole ratio, the *P*_f_ values start to increase with the rising curcumin concentration. The attained *P*_f_ values are approximately 74, 78, and 82 μm/s for mole ratios of 30:1, 10:1, and 4:1, respectively.

Figure 2B displays the water permeability values for DOPC/Chol at a 4/1 mol ratio with varying concentrations of curcumin. As the concentration of curcumin increases, the water permeability decreases, exhibiting a trend qualitatively similar to that observed with DOPC in the low-concentration region. In contrast to DOPC, we did not observe a concentration-dependent biphasic effect in the case of DOPC/Chol. Instead, we observed a gradual decrease in *P*_f_ values at all concentrations of curcumin tested with this trend leveling off as concentrations increased. It is noted that the water permeability of the control for DOPC/Chol at a 4/1 mol ratio (70 μm/s) is lower compared to that of pure DOPC in the absence of Chol (74 μm/s). This would be a consequence of the well-known condensing effect of Chol in PC lipid bilayers, as documented in previous studies,^45,46^ resulting in a reduction in the area occupied by the lipid and a subsequent reduction in water permeation.^47^

In general, water permeability has been demonstrated to be influenced by the physical characteristics of lipid bilayers, such as thickness, area per molecule, overall membrane fluidity, and rigidity,^47,48^ which are typically associated with the packing density of lipids.^49^ Hence, it is not unreasonable to infer that water permeability is a direct function of a membrane’s degree of order, with higher water permeability indicating a greater degree of membrane disorder. Our findings demonstrate differential behavior in water permeability. Curcumin at low concentrations (up to a lipid:curcumin mole ratio of 50:1) decreases water permeability in DOPC lipid bilayers, which could be indicative of a rigidifying effect of curcumin. Conversely, at high concentrations (above 50:1), there is an increase in the water permeability, possibly implying a fluidifying effect. In contrast, in cholesterol-enriched DOPC bilayers, a decrease in water permeability is observed without a significant dependence on curcumin concentration.

Our water permeability results align qualitatively with previous studies, in that they reveal a concentration-dependent impact of curcumin on lipid membranes. Research has shown that curcumin’s binding site within the lipid membrane varies with its concentration. At low concentrations (<3 mol %), curcumin primarily binds to the DOPC membrane interface, while at higher concentrations, it gradually incorporates into the acyl chain region in a transbilayer orientation. Solid-state NMR studies have demonstrated that curcumin enhances the overall segmental ordering of DMPC membranes. This effect is more pronounced at low concentrations (0.25–0.5%), while at higher concentrations (≥1%), curcumin forms oligomeric structures, reducing membrane thickness and molecular order compared to lower-concentration samples.^30^ Additionally, ^1^H NMR diffusometry of planar DMPC and DOPC bilayers has shown that the translational mobility of lipids varies with the curcumin concentration. Low concentrations (up to 7–8 mol %) lead to a moderate decrease in the lateral diffusion coefficient, while higher concentrations have no significant effect.^28^

Our findings regarding a concentration-dependent effect on water permeability can be explained by previous reports of the binding mode and its impact on the membrane. Previous studies have suggested a two-state binding of curcumin to lipid bilayers based on changes in membrane thickness and area upon curcumin binding to DOPC. Initially, curcumin binds to the water-membrane interface, and at higher concentrations, it gradually partitions into the hydrocarbon region of the bilayer.^50^ Combining X-ray diffraction with molecular dynamics (MD) computer simulations, researchers have reported that the presence of curcumin in the lipid headgroups decreases fluidity, whereas curcumin in the bilayer region results in the fluidizing of lipid membranes.^23^ The contrasting changes in water permeability (decrease at low concentrations and increase at high concentrations) reflect modulation of the physical properties of the lipid bilayer. At low concentrations (at and below a 50:1 lipid:curcumin mol ratio), curcumin may bind to the membrane–water interface region, reducing fluidity and sterically hindering the transport of water molecules. In contrast, at high concentrations (above a 50:1 lipid to curcumin mole ratio), curcumin may penetrate further into the acyl chain region of the lipid bilayer, creating disorder, disturbing the lipid bilayer’s packing order, and resulting in increased water permeability. MD simulations have shown that curcumin readily inserts into DOPC, leading to membrane thinning and an increase in area per lipid,^27^ which aligns with our observation of increased water permeability at high concentrations. Additionally, polarity profiles of DMPC and DSPC by EPR spin labeling technique indicate that curcumin at 10 mol % increases water penetration at the polar headgroups as well as in the hydrocarbon region of the bilayer;^29^ such phenomenon is likely associated with increased water permeability. It is important to note that an exact comparison of the concentration range at which the biphasic effect is observed is not possible due to differences in the lipid composition and experimental techniques employed.

In the presence of cholesterol, our data demonstrate a modest reduction in the water permeability without apparent concentration-dependent behavior. Previous reports have shown that curcumin partitions to the nonpolar region of the lipid bilayer of PC containing cholesterol (20 and 40%), as indicated by fluorescence spectroscopy.^51^ Our data, showing a curcumin-induced decrease in water permeability of the DOPC-cholesterol bilayer, are consistent with reports that curcumin enhances ordering in the DMPC acyl chains in the presence of cholesterol, as observed through ^2^H and ^14^N nuclear magnetic resonance spectroscopy.^52^

### Effect of Curcumin on Thermotropic Properties of the DOPC and DOPC/Chol Membranes

**Figure 3:**
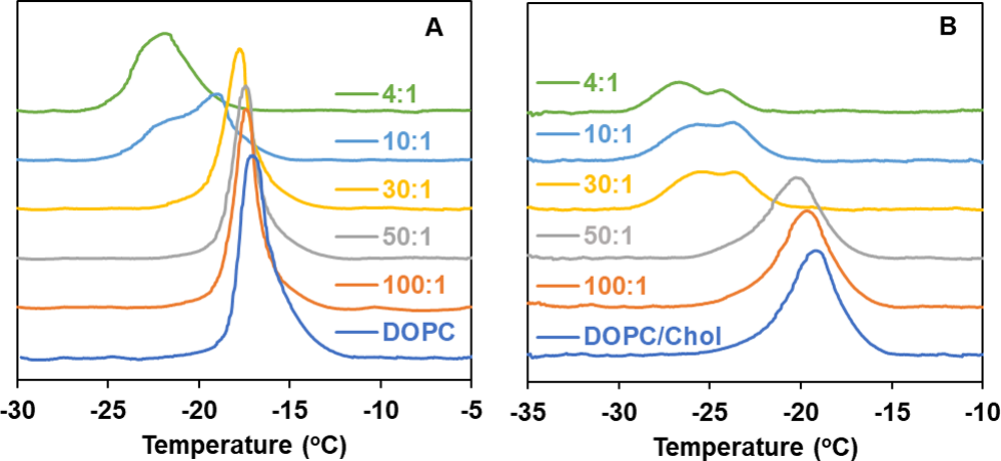
Endothermic calorimetric thermograms of (A) DOPC and (B) DOPC/Chol (4/1 mol ratio) MLVs containing different curcumin concentrations.

Figure 3 displays the endothermic DSC thermograms for phase transitions of MLVs of DOPC and DOPC/Chol (4/1 mol ratio) in the absence and presence of curcumin. The corresponding thermodynamic data are listed in Table 1. The control DOPC MLVs (no curcumin) in Figure 3A exhibit well-defined endothermic transitions with *T*_m_ = −16.98 °C with an enthalpy of 9.01 kcal/mol. These transitions are associated with the conversion of the lamellar gel phase L_β_ to the lamellar liquid-crystalline state L_α_ and are consistent with literature data (*T*_m_ = −18.3 ± 3.6 °C, Δ*H*= 9.0 ± 2.8 kcal/mol).^53^ The thermograms of DOPC MLVs are seen to be influenced by the inclusion of curcumin as a function of its concentration. At low concentrations of curcumin (100:1 mol ratio of DOPC to curcumin), *T*_m_ decreases slightly by 0.4 °C compared to the control, with a small reduction in Δ*H*. As the concentration of curcumin increases, up to a 30:1 mol ratio, only a gradual decrease in these values is observed (*T*_m_ decreases by 0.86 °C, and Δ*H* decreases by 17% compared to the control values). However, when curcumin is introduced at a 10:1 mol ratio, a splitting of the peak into two components with different peak temperatures (at −19.21 and −22.08 °C) is observed. This splitting likely indicates phase separation due to an uneven curcumin distribution in the lipid bilayer, resulting in curcumin-poor and curcumin-rich regions. Peak deconvolution (Figure S1 in the Supporting Information) reveals that the lower temperature shoulder component (curcumin-rich region) continues to grow from 35% at a 10:1 mol ratio to 82% at a 4:1 mol ratio. The enthalpy of the transition (total area of both peaks) gradually decreases with increasing curcumin concentrations, from 9.01 to 6.24 kcal/mol (about a 30% reduction compared to the control value) at the highest concentrations (4:1 mol ratio) tested.

**Table 1:** Thermodynamic Parameters (*T*_m_ and Δ*H*) for Main Phase Transition of MLVs of DOPC, and DOPC with Chol, with Different Curcumin Concentrations

lipid	DOPC			DOPC/Chol (4/1 mol ratio)		
lipid: curcumin (mol ratio)	*T*_m_ (°C)	Δ*H* (kcal/mol)	second peak, *T*_m_^a^ (rel area %)	*T*_m_ (°C)	Δ*H* (kcal/mol)	second peak, *T*_m_^a^ (rel area %)
1:0	–16.98 ± 0.18	9.01 ± 0.25		–19.37 ± 0.12	2.81 ± 0.34	
100:1	–17.39 ± 0.05	8.76 ± 0.38		–19.80 ± 0.07	2.36 ± 0.10	
50:1	–17.48 ± 0.05	7.52 ± 0.24		–20.39 ± 0.06	2.16 ± 0.26	
30:1	–17.84 ± 0.03	7.46 ± 0.20		–23.41 ± 0.09	1.49 ± 0.10	–26.00 (56%)
10:1	–19.21 ± 0.13	6.40 ± 0.15	–22.08 (35%)	–23.45 ± 0.13	1.48 ± 0.10	–26.11 (60%)
4:1	–20.30 ± 0.75	6.24 ± 0.23	–22.28 (82%)	–24.08 ± 0.25	0.94 ± 0.15	–26.76 (72%)

a Curve-fitting simulations were performed using Origin software to deconvolute and fit into two components corresponding to curcumin-rich (lower *T*_m_) and curcumin-poor (higher *T*_m_) regions (Figure S1 in the Supporting Information). Enthalpy data represent the total area for all peaks.

The endothermic thermograms of MLVs of DOPC/Chol (4/1 mol ratio) are shown in Figure 3B. For the control bilayer (i.e., containing cholesterol but before the introduction of curcumin), there are significant changes in the thermogram relative to pure DOPC. The *T*_m_ decreased to −19.37 °C (from −16.98 °C for pure DOPC) with a reduction in Δ*H* to 2.81 kcal/mol (from 9.01 kcal/mol for pure DOPC), accompanied by an overall broadening of the peak. These changes are consistent with previously reported data.^54^ The addition of low concentrations of curcumin to a binary mixture of DOPC and Chol (100:1 lipid mixture to curcumin) follows a trend similar to that of pure DOPC: a mild decrease in *T*_m_ and Δ*H*, which is further reduced at a 50:1 mol ratio. However, starting at a 30:1 mol ratio, phase separation becomes apparent, indicating domain formation. Such putative domain formation is not apparent for pure DOPC until a higher concentration of curcumin is employed, specifically at and above the 10:1 mol ratio. With increasing curcumin concentrations (up to a 4:1 mol ratio), the relative proportion of the two components (curcumin-rich and curcumin-poor) shifts from the higher-temperature component (curcumin-poor) to the lower-temperature component (curcumin-rich). The area of the lower temperature component (*T*_m_= −26 °C) increased from 56% (at 30:1), 60% (at 10:1), to 72% (at 4:1 mol ratio), which we interpret as being the growth of a curcumin-rich domain at the expense of a curcumin-poor domain.

As shown in Figure 3, curcumin interacts with both DOPC and DOPC/Chol liposomes, influencing their thermotropic phase behavior in a concentration-dependent manner. Previous studies on liposomal thermotropic properties in the presence of curcumin have primarily focused on DMPC and DPPC. These studies have reported a decrease in *T*_m_ and the suppression of the enthalpy of the phase transition at curcumin concentrations of (respectively) up to 5 mol % with DMPC and DPPC,^30^ 2 mol % with DPPC,^21^ and up to approximately 16 mol % with DPPC.^25^ It is worth noting that our DSC experiment was conducted on a fluid-phase membrane, composed of unsaturated DOPC (*T*_m_= −17 °C), in contrast to the saturated acyl chain PC of DMPC (*T*_m_= 23 °C) and DPPC (*T*_m_= 41 °C). In our study, at low concentrations of curcumin (up to a 30:1 mol ratio), the main phase transition peaks were symmetrical with a modest decrease in *T*_m_ and Δ*H*, consistent with the findings reported for DMPC and DPPC,^30^ indicating a homogeneous distribution of curcumin in the bilayer and minimal perturbation of the acyl chain packing. However, at higher concentrations, evidence of phase separation becomes apparent. A similar phase separation has been reported at a comparable DPPC to curcumin molar ratio (16:1).^25^ Heterogeneous distribution of curcumin and the formation of segregated domains have also been reported for DMPC MLVs at >1 mol %, whereas a homogeneous distribution is shown at ≤1 mol %.^22^ Our data, showing the increasing contribution from curcumin-rich domains (from 35 to 82% at 10:1 to 4:1 mol ratios, respectively, for DOPC membranes), indicate a different binding mode in the lipid bilayer, consistent with the concentration-dependent biphasic effect observed in water permeability. The thermotropic effects of curcumin on DOPC/Chol lipid bilayers equally demonstrate a strong perturbation of the lipid environment, as evidenced by the magnitude of the reduction in *T*_m_ and Δ*H*. As curcumin concentrations increased, it is likely that interactions with the membrane became saturated due to limited free space in the acyl chain region (cholesterol’s predominant location). This saturation was evidenced by phase separation appearing on the thermogram at a 30:1 lipid:mol ratio, whereas a higher curcumin concentration was needed to observe a similar phase separation in pure DOPC (10:1 mol ratio). This suggests curcumin’s ability to interact with and perturb the thermodynamic properties of DOPC/Chol membranes. Overall, for both DOPC and DOPC/Chol lipid membranes, a modest decrease in *T*_m_ and Δ*H* at low curcumin concentrations indicates relatively unperturbed acyl chain packing of the membrane environment, implying binding at the lipid membrane surface. In contrast, the prominent phase separation observed at high concentrations signals curcumin’s ability to likely penetrate into the acyl chain environment, providing additional evidence for a concentration-dependent binding mode of curcumin, consistent with our water permeability data described in the previous section.

### Effects of Curcumin on Conformational Changes of the DOPC and DOPC/Chol Membranes

ATR-FTIR was employed to further examine the interaction of curcumin with the DOPC and DOPC/Chol membranes. Phospholipid membranes have three major regions with infrared-active groups: the lipid acyl chain, interfacial carbonyl, and phosphate headgroup. These regions can reveal structural and organizational changes when interacting with membrane-active molecules.^55,56^ The acyl chain region consists of symmetric (around 2850 cm^–1^) and antisymmetric (around 2920 cm^–1^) stretching vibration bands of CH_2_. This region is sensitive to gauche–trans conformational changes in lipid acyl chains and is used to monitor information associated with the overall degree of ordering and packing of the lipid bilayer during interactions with membrane-active molecules. The interfacial carbonyl stretching vibration region (C=O around 1724–1742 cm^–1^) provides insights into the hydration state and/or hydrogen bonding to the ester carbonyl groups.^57^ The phosphate headgroup region (PO_2_^–^ symmetric and antisymmetric stretching vibration band around 1000–1240 cm^–1^) suggests information about hydration and hydrogen bonding at the surfaces of hydrated phospholipid assemblies.^58^ The characteristics of these IR bands and their changes in wavenumber and bandwidth reflect the structural and dynamic alterations in the lipid bilayer. These changes have been used for a molecular-level view of interactions with membrane-active molecules.

**Figure 4:**
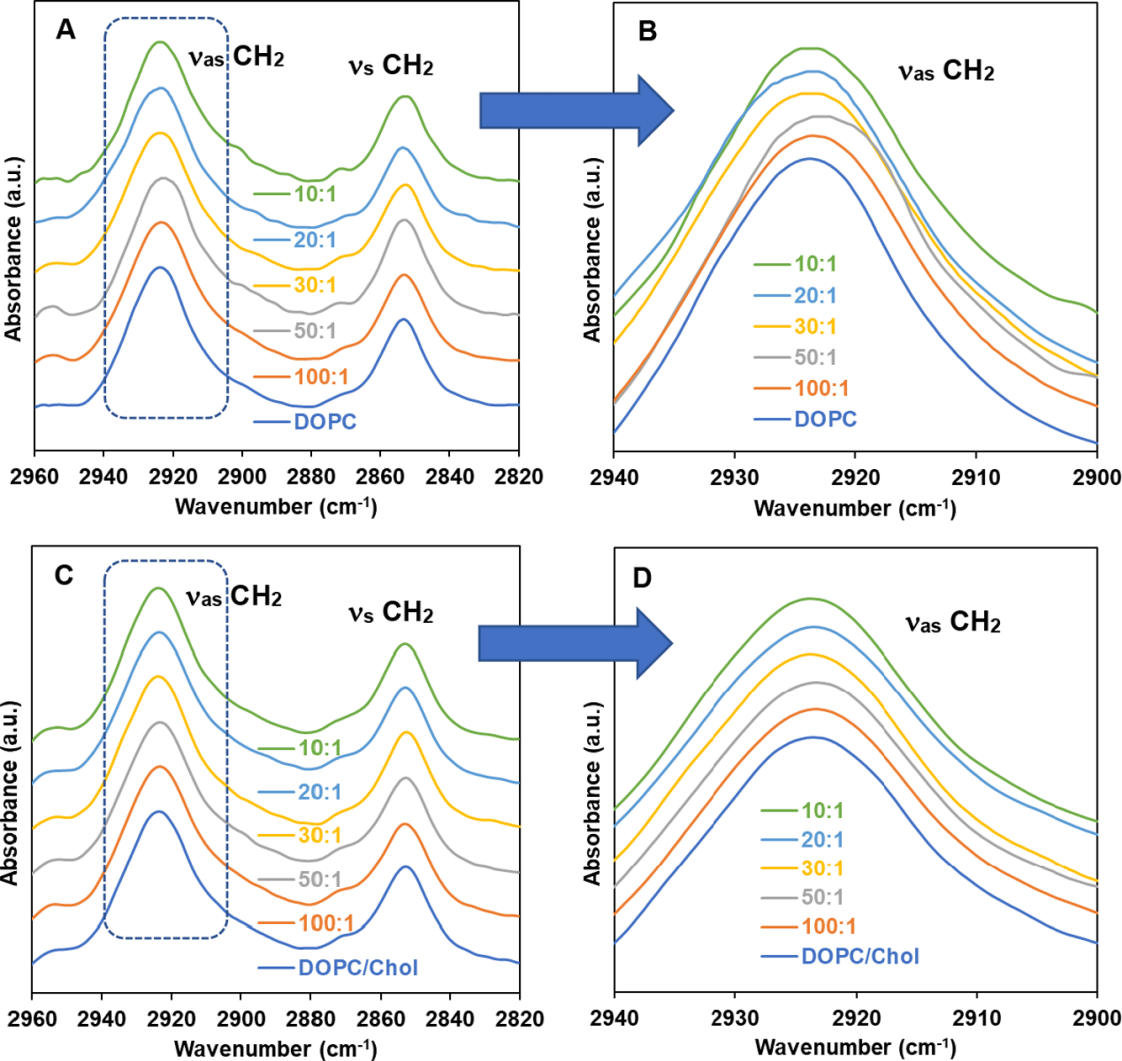
Representative ATR-IR spectra in the stretching vibration of the acyl chain CH_2_ groups of (A) DOPC and (C) DOPC with Chol (4/1 mol ratio) membranes, with varying mol ratios of curcumin at 25 °C, with the inset representing expanded regions of interest of ν_as_ CH_2_ (B, D) showing wavenumber shifts more readily. The spectra are normalized using the respective CH_2_ antisymmetric stretching vibration band at around 2920 cm^–1^ and vertically shifted.

Figure 4 displays ATR-FTIR in the CH_2_ region (2800–3000 cm^–1^) for membranes of DOPC and DOPC containing cholesterol (4/1 mol ratio), in the presence of varying concentrations of curcumin at 25 °C. An expanded region of ν_as_ CH_2_ (Figure 4B,D) is also shown in Figure 4 (dotted box in Figure 4A,C). There was no observable change in the symmetric stretching (ν_s_ CH_2_) part of the spectra. As seen in Figure 4A,B, our IR band analysis shows slight but noticeable wavenumber shifts (∼1 cm^–1^) in the CH_2_ antisymmetric stretching region: a red shift at low curcumin concentrations (up to 50:1) and a blue shift at higher concentrations (above 50:1 mol ratios of DOPC to curcumin). The red shift in ν_as_ CH_2_ indicates an increase in the order of the membranes, while the blue shift reflects an increase in gauche conformers and reduced acyl chain order, implying a more disordered state.^55^ It is interesting to note that the concentration-dependent biphasic wavenumber shifts in ν_as_ CH_2_ are consistent with what we have observed in the change of the water permeability coefficient (*P*_f_): *P*_f_ decreases at low concentrations (up to 50:1) and increases at high concentrations (above 50:1). Note that DOPC is in a fluidic state (*T*_m_ = −17 °C) at ambient temperature, which corresponds to a high degree of freedom and conformational flexibility. This leads to a low degree of lateral interactions, and any change in the acyl chain conformational order in fluidic membranes is relatively less pronounced than in lipids with acyl chains going through acyl chain-melting phase transitions (e.g., DPPC). The fluidic character of the DOPC membranes is presumably the reason for its relatively small wavenumber shifts.

The bandwidth of the CH_2_ stretching modes reflects changes in the mobility of the acyl chains, thereby providing information about the dynamics of membrane systems. Our spectra show a slight increase in the overall bandwidth of CH_2_ antisymmetric stretching vibration modes (ν_as_ CH_2_) in DOPC (Figure S2 in the Supporting Information) in the presence of high concentrations of curcumin (from 21.67 cm^–1^ for DOPC to 23.35 cm^–1^ for DOPC/curcumin 10/1 mol ratio). This indicates increased dynamics of acyl chains at high curcumin concentrations, a sign of increased fluidity in the membrane system. On the other hand, for cholesterol-containing DOPC membranes (4/1 PC/Chol mol ratio), there are no observable wavenumber shifts (Figure 4C,D) or bandwidth changes (Figure S2, from 24.27 to 24.09 cm^–1^), indicating no significant changes in lipid acyl chain conformation.

**Figure 5:**
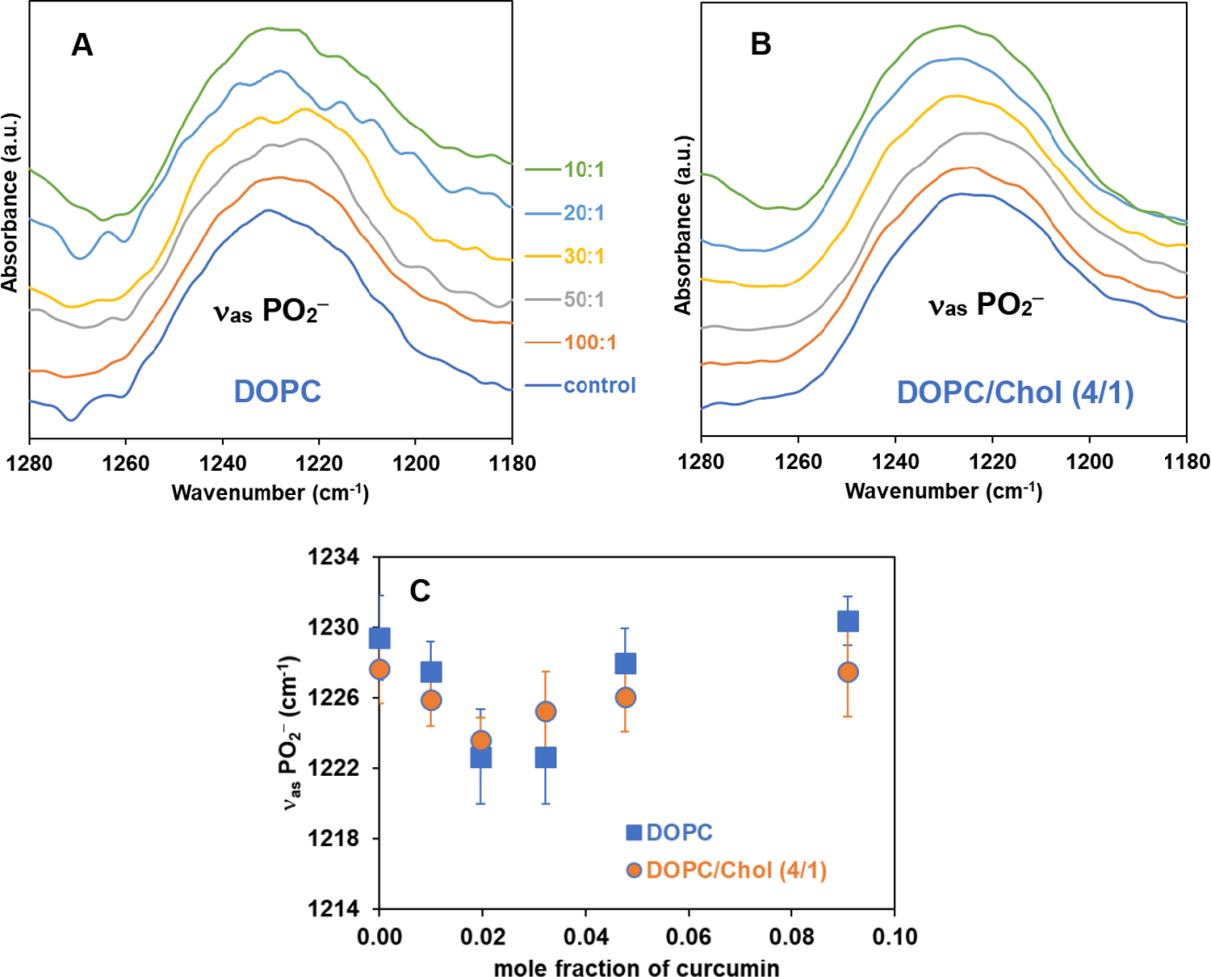
Representative ATR-IR spectra of (A) DOPC, (B) DOPC with Chol (4–1 mol ratio) membranes, and (C) plot of wavenumber with varying concentrations of curcumin in the region of antisymmetric PO_2_^–^ stretching vibration bands. Each spectrum is recorded at 25 °C. The spectra are normalized using the CH_2_ antisymmetric stretching vibration band at 2920 cm^–1^ and vertically shifted.

Figure 5A,B shows the phosphate PO_2_^–^ antisymmetric region as a function of curcumin concentrations for both pure DOPC, and DOPC with Chol (4/1 mol ratio), respectively. In addition, the wavenumbers of these stretching modes are also plotted in Figure 5C (the value of the wavenumber is shown in the Supporting Information, Table S2). It has been reported that the PO_2_^–^ antisymmetric vibration band is more sensitive to environmental influences than the symmetric PO_2_^–^.^55^

For both DOPC and DOPC with cholesterol, noticeable changes do occur in this region. In the case of DOPC membranes (Figure 5A,C), the ν_as_ PO_2_^–^ decreases by 2 cm^–1^ upon the introduction of curcumin at a 100:1 mol ratio. A continued decrease in ν_as_ PO_2_^–^ is observed with the increased addition of curcumin, resulting in a red shift of 6 cm^–1^ at 30:1 lipid to curcumin concentrations. However, at higher curcumin concentrations (at 20:1 and 10:1), the opposite trend is observed, with a blue shift in ν_as_ PO_2_^–^ (Figure 5A,C). It has been well-established that changes in the wavenumber values of ν_as_ PO_2_^–^ represent the hydration profile of phospholipid head groups.^58^ A decrease (red shift) in the wavenumber of ν_as_ PO_2_^–^ may indicate that curcumin promotes the formation of hydrogen bonds with the phosphate groups when at concentrations up to 30:1 mol ratios, while the opposite occurs at higher concentrations. Qualitatively similar trends in wavenumber shifts of ν_as_ PO_2_^–^ are observed for DOPC containing cholesterol, with somewhat less pronounced shifts as compared to DOPC membranes, as shown in Figure 5B,C: curcumin induces a red shift in wavenumber at low concentrations. On the other hand, at high concentrations of curcumin, a shift in wavenumber to higher values (blue shift) is seen, indicative of dehydration around the headgroup region. Recall that our DSC thermograms show evidence of phase separation at curcumin concentrations similar to the change in wavenumber shift observed for the ν_as_ PO_2_^–^ peak for both DOPC and DOPC with Chol. As for peaks in the C=O stretching vibration region, its characteristics can provide information about the hydrogen bonds around the glycerol backbones of DOPC, but its broad band around ∼1738 cm^–1^ in this region is too complex to be clearly analyzed. In sum, the characteristic changes of vibrational modes in both CH_2_ and PO_2_^–^ regions provide evidence that curcumin interacts with the polar headgroup region of the lipids and influences the conformation of the lipid assembly in a concentration-dependent biphasic manner, consistent with our data from water permeability and DSC.

## Conclusions

**Figure 6:**
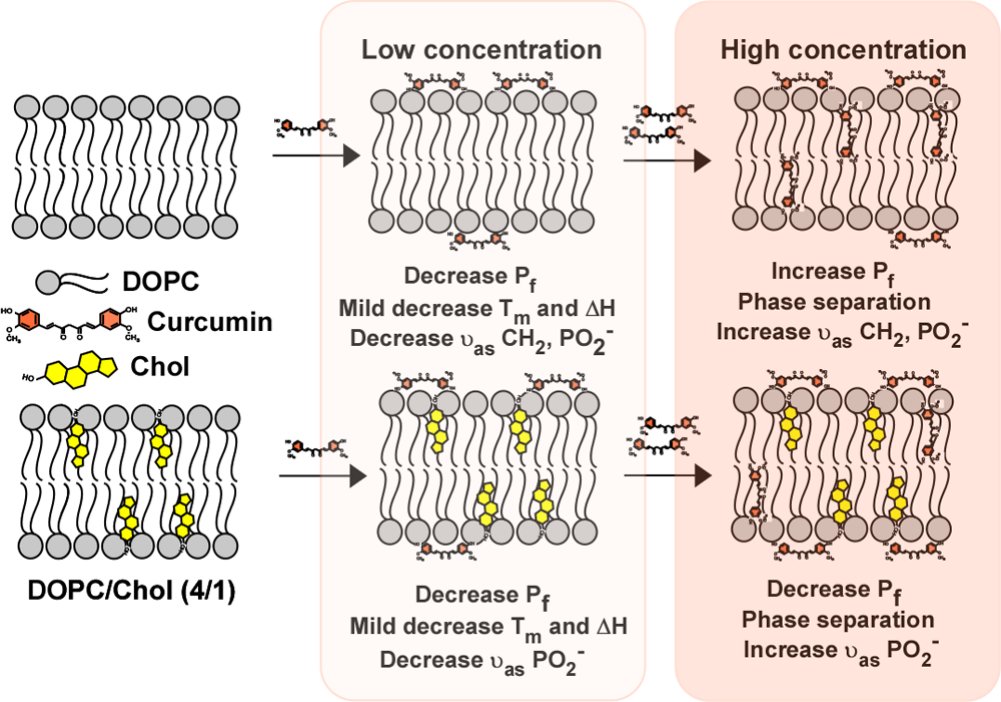
Schematic representation of curcumin interaction with DOPC lipid bilayer in the absence and presence of cholesterol illustrating concentration-dependent effect (not drawn to scale).

Growing data point to an important role for nonspecific interactions of biologically active molecules with lipid membranes in modulating the functions of membrane proteins. Based on the findings in this study, curcumin modifies membrane properties, as evidenced by water permeabilities determined via a DIB platform, thermotropic properties, determined by DSC, and conformational changes using ATR-FTIR. A schematic representation summarizing our results is shown in Figure 6.

Our study reveals that curcumin’s interaction with fluidic DOPC membranes alters the lipid bilayer’s barrier properties. Specifically, we observed a decrease in water permeability at low curcumin concentrations (up to 2 mol % of curcumin) and an increase in water permeability at and above ∼3 mol % of curcumin. These findings were probed by using a DIB-based water transport study. At low concentrations, curcumin is believed to bind to the lipid–water interface through hydrogen bonding with the phosphate headgroup. This binding would reduce membrane fluidity and sterically hinder the transport of water molecules, resulting in decreased water permeability. Conversely, at high concentrations, curcumin may penetrate into the acyl chain region, fluidizing the membrane and increasing the water permeability. Our DSC studies show that the incorporation of curcumin into DOPC membranes has a mild effect on decreasing *T*_m_ and reducing Δ*H* at low concentrations. However, at high curcumin concentrations (10 mol % or greater), evidence of phase separation emerged, indicating a heterogeneous membrane environment and increased disruption of the acyl chain environment. These findings align with those of our water permeability studies. ATR-FTIR studies demonstrated a red shift (for antisymmetric CH_2_ stretch) at low curcumin concentrations and a blue shift at high concentrations, along with peak broadening in the CH_2_ antisymmetric stretching vibration modes of the oleoyl chains. These observations reflect a concentration-dependent biphasic effect: an increase in the order at low concentrations and an increase in the percentage of disorder in the acyl chain of DOPC membranes at high concentrations. Furthermore, the PO_2_^–^ antisymmetric stretching vibration band exhibited a similar effect. Curcumin shifted the maximum of the absorption band toward lower wavenumbers at low concentrations, but this shift was reversed at high concentrations. This indicates that curcumin alters the conformation of the polar part of DOPC depending on its concentration, strengthening hydrogen bonding with the PO_2_^–^ group at low concentrations and having the opposite effect at high concentrations. Similar qualitative effects were seen in the presence of cholesterol, demonstrating curcumin’s ability to interact with cholesterol-containing DOPC, but with a less pronounced concentration effect.

In summary, our comprehensive studies from various techniques provide clear evidence of curcumin’s concentration-dependent biphasic effect on water transport phenomena, thermal properties, and structural characteristics of membranes. These findings indicate distinct modes of interaction and impacts by curcumin on the membrane, whether cholesterol is present or not, in fluid DOPC membranes. Based on our experimental findings that demonstrate curcumin’s ability to modify the biophysical properties of lipid membranes, it is essential to consider the membrane-mediated protein modification pathway when exploring the potential therapeutic effects of curcumin. This broader perspective is crucial for a comprehensive understanding of the mechanisms underlying its bioactivity and successful translation into effective medicine. The interactions with the bilayer are expected to modify the energetic landscape of membrane-embedded receptor proteins. The modes of interactions we have found can also be useful in providing insights when designing drug delivery platforms loaded with curcumin that have optimal formulation parameters.

## Experimental Section

### Sample Preparations

1,2-Dioleoyl-*sn*-glycero-3-phosphocholine (DOPC) in chloroform was obtained from Avanti Polar Lipids (Alabaster, AL) and used as received. All other chemicals, including cholesterol (Chol) and curcumin, were purchased from Sigma-Aldrich and used without additional purification. Squalene (SqE, 2,6,10,15,19,23-hexamethyl-2,6,10,14,18,22-tetracosahexaene; C_30_H_50_) was used as the immiscible organic phase for creating a DIB for water permeability experiments. SqE was chosen as an immiscible organic phase as it is known to be excluded from the bilayer, forming a solvent-free DIB.^59,60^ Lipid and cholesterol samples were stored at −20 °C, while SqE was kept within the temperature range of 2–8 °C. To prepare an oil solution containing DOPC (with or without cholesterol), we evaporated the chloroform solution of DOPC (optionally with cholesterol) under argon gas to form a dried thin film of lipid or lipid mixture. This film was then vacuum-dried overnight to ensure the complete removal of any residual solvent. Curcumin stock solution (prepared using chloroform/methanol, 8/2, v/v) was mixed with a solution of lipid or lipid mixture, following which the volatile solvent was evaporated entirely, yielding a dried curcumin/lipid film with a defined mol ratio. In order to prevent photodegradation in samples containing curcumin or DOPC, all sample containers were wrapped with aluminum foil to avoid extended light exposure. Solutions containing curcumin or DOPC were freshly prepared immediately before each experiment, and new sets of samples were prepared daily for repeated experiments. For water permeability experiments, the dried curcumin/lipid film was dissolved in SqE to a level of total lipid concentration of 5 mg/mL. In the case of samples containing cholesterol, a 4/1 mol ratio of DOPC/Chol mixtures was used. For the DSC and ATR-FTIR experiment, the dried curcumin/lipid films were subsequently rehydrated with pure water to achieve a total lipid concentration of approximately 16 mg/mL for DSC and around 80 mg/mL for ATR-FTIR. The mixtures were vigorously vortexed for about 5 min to obtain a suspension of multilamellar vesicles (MLVs) and then subjected to approximately 30 min of bath sonication to reach equilibrium. Aqueous solutions containing osmolytes (NaCl at nominally 0.1 M) were prepared from deionized water (18.2 MΩ cm) purified using a Millipore water system (Direct Q-3). The osmolality (in mOsm/kg) of all aqueous solutions was measured using a vapor pressure osmometer (VAPRO model 5600) immediately after each solution’s fresh preparation and before its utilization.

### Water Permeability

**Figure 7:**
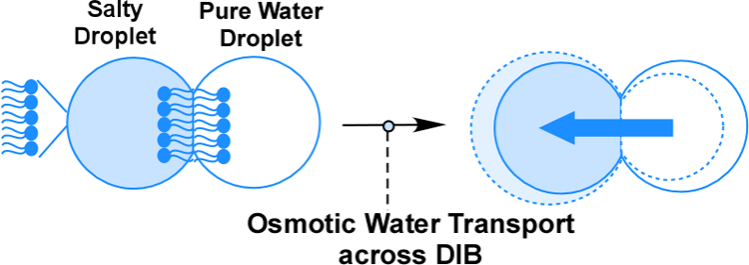
General schematic of the DIB-based osmotic water permeability experiment. When an osmotic pressure imbalance exists between two adhering aqueous droplets in a DIB, water transport occurs through the droplet bilayer, leading to a noticeable change in the droplet diameter. We monitor and record osmotic water transport for approximately 5 min. The starting size of each aqueous microdroplet (solid line) is typically in the range of ∼100 μm in diameter. A blue arrow represents the direction of the water transport.

The measurement of water permeability was conducted by using a model membrane formed through the DIB method. The detailed experimental setup and procedures have been described elsewhere.^38^ The experimental platform comprises two hydraulic micropipet manipulators (Narishige) for the generation and control of aqueous microdroplets. Glass micropipets controlled by the manipulators dispense droplets into a pool of oil held between the glass slides. An inverted microscope (Nikon Eclipse Ti–S with a halogen lamp) is employed for observation of the microdroplets. This setup is supported on a vibration-isolated workstation (Newport) and is equipped with a camera (Andor Zyla sCMOS) directly attached to the microscope for real-time recording of the generated microdroplets and their size changes. Figure 7 provides a general schematic of the DIB-based osmotic water permeability measurement. A pair of osmotically unbalanced aqueous droplets are created, typically one being pure water and the other being a droplet of 0.1 M NaCl, each with a diameter of about 100 μm. These droplets are suspended in SqE solvent, which contains lipid or lipid mixtures with varying concentrations of curcumin. The solid line in Figure 7 represents the original size of two aqueous droplets, and the dashed line represents the final size, depicting one swollen and one shrunken droplet as a result of water movement (direction of water transport shown in blue arrow). All water permeability experiments in this study were conducted at 30 °C, using a custom-built temperature-controlled microchamber that was thermostated via an external circulating water bath. The temperature of the microchamber containing lipid mixtures is measured by a thermocouple wire and is accurate to ±0.1 °C. The detailed method for calculating water permeability is provided in the Supporting Information.

### Differential Scanning Calorimetry (DSC)

Thermal phase transition studies were conducted using a TA Q2000 differential scanning calorimeter, and the data were analyzed with TA Universal Analysis software to investigate the main phase transition behavior of the samples. The main phase transition temperature, *T*_m_, corresponds to the temperature at the apex of the endothermic transition peak. The phase transition enthalpy was determined by integrating the area under the heat capacity curve. Aliquots of approximately 15 μL of the MLVs prepared as described in the Experimental Section were hermetically sealed and subjected to heating and cooling at rates of 5 °C/min, ranging from −40 to 0 °C, with a high-purity nitrogen flow rate of 50 mL/min. Reported values represent the average of three separately prepared samples. Each sample underwent three cycles of heating and cooling, and in all cases, reproducible results were obtained with no hysteresis.

### Attenuated Total Reflection-Fourier Transform Infrared Spectroscopy (ATR-FTIR)

All ATR-FTIR spectra were acquired by using a Thermo Scientific Nicolet iS20 spectrometer equipped with a deuterated triglycine sulfate (DTGS) detector. For measurements, we utilized a GladiATR single-reflection ATR accessory with a diamond crystal (Pike Technologies, USA) and a temperature-controlled plate. Approximately 40 μL of the MLVs prepared as described in the relevant portion of the Experimental Section was spread onto the diamond crystal surface using an ATR liquid retainer and volatiles cover accessory (Pike Technologies, USA). Spectra were recorded in the 400–4000 cm^–1^ region at 25 °C, with 200 scans accumulated and a spectral resolution of 4 cm^–1^. All reported IR spectra were obtained after subtraction of background spectra measured with deionized water, which were recorded before each new sample. The diamond crystal, ATR liquid retainer, and volatile cover were meticulously cleaned with isopropanol after each sample and allowed to dry before the next set of measurements. Three separate sets of samples were prepared, and each sample was scanned 3–5 times for consistency.

### Data Analysis

Water permeability data represent an average of individual permeability runs (*n* ≥ 20), and standard deviation as error bars (mean ± standard deviation). The recorded videos and images were postanalyzed to measure the dimension of droplets and contact area using custom-built image analysis software. DSC and ATR-FTIR data represent the average of three separately prepared samples and are expressed as mean ± standard deviation. For the DSC thermogram, the data were analyzed with TA Universal Analysis software to investigate the main phase transition behavior of the samples. Additionally, baseline subtraction and curve-fitting simulations were performed using OriginPro 9.7 software to deconvolute and fit into two components corresponding to higher *T*_m_ and lower *T*_m_ regions. For ATR-FTIR, data processing was performed using the OMNIC 9 (Thermo) and ProtaCAL (BioTools) software packages.

## Supporting Information Available

Analysis of water permeability data; water permeability coefficient for DOPC and DOPC/Chol (4/1 mol/mol) membrane, as a function of curcumin concentrations; DSC thermograms with curve-fitting simulations; ATR-IR spectra of CH_2_ stretching vibrational modes; and wavenumber with varying concentrations of curcumin in the region of antisymmetric PO_2_^–^ stretching vibration bands (PDF)

## References

[ref1] Hatcher H.; Planalp R.; Cho J.; Torti F.; Torti S. Curcumin: from ancient medicine to current clinical trials. Cell. Mol. Life Sci. 2008, 65, 1631–1652. 10.1007/s00018-008-7452-4. 18324353 PMC4686230

[ref2] Hu S.; Maiti P.; Ma Q.; Zuo X.; Jones M. R.; Cole G. M.; Frautschy S. A. Clinical development of curcumin in neurodegenerative disease. Expert Rev. Neurother. 2015, 15 (6), 629–637. 10.1586/14737175.2015.1044981. 26035622 PMC6800094

[ref3] Kunnumakkara A. B.; Hegde M.; Parama D.; Girisa S.; Kumar A.; Daimary U. D.; Garodia P.; Yenisetti S. C.; Oommen O. V.; Aggarwal B. B. Role of Turmeric and Curcumin in Prevention and Treatment of Chronic Diseases: Lessons Learned from Clinical Trials. ACS Pharmacol. Transl. Sci. 2023, 6 (4), 447–518. 10.1021/acsptsci.2c00012. 37082752 PMC10111629

[ref4] Nelson K. M.; Dahlin J. L.; Bisson J.; Graham J.; Pauli G. F.; Walters M. A. The essential medicinal chemistry of curcumin: miniperspective. J. Med. Chem. 2017, 60 (5), 1620–1637. 10.1021/acs.jmedchem.6b00975. 28074653 PMC5346970

[ref5] Salehi B.; Stojanović-Radić Z.; Matejić J.; Sharifi-Rad M.; Kumar N. V. A.; Martins N.; Sharifi-Rad J. The therapeutic potential of curcumin: A review of clinical trials. Eur. J. Med. 2019, 163, 527–545. 10.1016/j.ejmech.2018.12.016. 30553144

[ref6] Zhou H.; S Beevers C.; Huang S. The targets of curcumin. Curr. Drug Targets 2011, 12 (3), 332–347. 10.2174/138945011794815356. 20955148 PMC3025067

[ref7] Heger M.; van Golen R. F.; Broekgaarden M.; Michel M. C. The molecular basis for the pharmacokinetics and pharmacodynamics of curcumin and its metabolites in relation to cancer. Pharmacol. Rev. 2014, 66 (1), 222–307. 10.1124/pr.110.004044. 24368738

[ref8] Ingolfsson H. I.; Koeppe R. E.; Andersen O. S. Curcumin is a modulator of bilayer material properties. Biochemistry 2007, 46 (36), 10384–10391. 10.1021/bi701013n. 17705403

[ref9] Ingólfsson H. I.; Thakur P.; Herold K. F.; Hobart E. A.; Ramsey N. B.; Periole X.; de Jong D. H.; Zwama M.; Yilmaz D.; Hall K.; et al. Phytochemicals perturb membranes and promiscuously alter protein function. ACS Chem. Biol. 2014, 9 (8), 1788–1798. 10.1021/cb500086e. 24901212 PMC4136704

[ref10] Baell J.; Walters M. A. Chemistry: Chemical con artists foil drug discovery. Nature 2014, 513 (7519), 481–483. 10.1038/513481a. 25254460

[ref11] Hopkins A. L.; Groom C. R. The druggable genome. Nat. Rev. Drug Discovery 2002, 1 (9), 727–730. 10.1038/nrd892. 12209152

[ref12] Sriram K.; Insel P. A. G protein-coupled receptors as targets for approved drugs: how many targets and how many drugs?. Mol. Pharmacol. 2018, 93 (4), 251–258. 10.1124/mol.117.111062. 29298813 PMC5820538

[ref13] Corradi V.; Sejdiu B. I.; Mesa-Galloso H.; Abdizadeh H.; Noskov S. Y.; Marrink S. J.; Tieleman D. P. Emerging diversity in lipid–protein interactions. Chem. Rev. 2019, 119 (9), 5775–5848. 10.1021/acs.chemrev.8b00451. 30758191 PMC6509647

[ref14] Escribá P. V.; González-Ros J. M.; Goñi F. M.; Kinnunen P. K.; Vigh L.; Sánchez-Magraner L.; Fernández A. M.; Busquets X.; Horváth I.; Barceló-Coblijn G. Membranes: a meeting point for lipids, proteins and therapies. J. Cell. Mol. Med. 2008, 12 (3), 829–875. 10.1111/j.1582-4934.2008.00281.x. 18266954 PMC4401130

[ref15] Halbleib K.; Pesek K.; Covino R.; Hofbauer H. F.; Wunnicke D.; Hänelt I.; Hummer G.; Ernst R. Activation of the unfolded protein response by lipid bilayer stress. Mol. Cell 2017, 67 (4), 673–684. 10.1016/j.molcel.2017.06.012. 28689662

[ref16] Brown M. F. Modulation of rhodopsin function by properties of the membrane bilayer. Chem. Phys. Lipids 1994, 73 (1–2), 159–180. 10.1016/0009-3084(94)90180-5. 8001180

[ref17] Prasanna P.; Upadhyay A. Flavonoid-based nanomedicines in Alzheimer’s disease therapeutics: Promises made, a long way to go. ACS Pharmacol. Transl. Sci. 2021, 4 (1), 74–95. 10.1021/acsptsci.0c00224. 33615162 PMC7887745

[ref18] Gayathri K.; Bhaskaran M.; Selvam C.; Thilagavathi R. Nano formulation approaches for curcumin delivery-a review. J. Drug Deliv Sci. Technol. 2023, 82, 104326 10.1016/j.jddst.2023.104326.

[ref19] Feng T.; Wei Y.; Lee R. J.; Zhao L. Liposomal curcumin and its application in cancer. Int. J. Nanomed. 2017, 6027–6044. 10.2147/ijn.s132434. PMC5573051 28860764

[ref20] Li L.; Braiteh F. S.; Kurzrock R. Liposome-encapsulated curcumin: in vitro and in vivo effects on proliferation, apoptosis, signaling, and angiogenesis. Cancer 2005, 104 (6), 1322–1331. 10.1002/cncr.21300. 16092118

[ref21] Sharma V. K.; Gupta J.; Srinivasan H.; Bhatt H.; García Sakai V.; Mitra S. Curcumin Accelerates the Lateral Motion of DPPC Membranes. Langmuir 2022, 38 (31), 9649–9659. 10.1021/acs.langmuir.2c01250. 35878409

[ref22] Sahu A. K.; Mishra A. K. Curcumin-induced membrane property changes in DMPC multilamellar vesicles and the effects of membrane-destabilizing molecules on curcumin-loaded multilamellar vesicles. Langmuir 2021, 37 (43), 12753–12766. 10.1021/acs.langmuir.1c02407. 34694126

[ref23] Alsop R. J.; Dhaliwal A.; Rheinstädter M. C. Curcumin protects membranes through a carpet or insertion model depending on hydration. Langmuir 2017, 33 (34), 8516–8524. 10.1021/acs.langmuir.7b01562. 28548854

[ref24] Hung W.-C.; Chen F.-Y.; Lee C.-C.; Sun Y.; Lee M.-T.; Huang H. W. Membrane-thinning effect of curcumin. Biophys. J. 2008, 94 (11), 4331–4338. 10.1529/biophysj.107.126888. 18310254 PMC2480666

[ref25] Pérez-Lara A.; Ausili A.; Aranda F. J.; de Godos A.; Torrecillas A.; Corbalan-Garcia S.; Gomez-Fernandez J. C. Curcumin disorders 1, 2-dipalmitoyl-sn-glycero-3-phosphocholine membranes and favors the formation of nonlamellar structures by 1, 2-dielaidoyl-sn-glycero-3-phosphoethanolamine. J. Phys. Chem. B 2010, 114 (30), 9778–9786. 10.1021/jp101045p. 20666521

[ref26] Ileri Ercan N. Understanding interactions of curcumin with lipid bilayers: a coarse-grained molecular dynamics study. J. Chem. Inf. Model. 2019, 59 (10), 4413–4426. 10.1021/acs.jcim.9b00650. 31545601

[ref27] Lyu Y.; Xiang N.; Mondal J.; Zhu X.; Narsimhan G. Characterization of interactions between curcumin and different types of lipid bilayers by molecular dynamics simulation. J. Phys. Chem. B 2018, 122 (8), 2341–2354. 10.1021/acs.jpcb.7b10566. 29394060

[ref28] Filippov A. V.; Kotenkov S. A.; Munavirov B.; Antzutkin O. N. Effect of curcumin on lateral diffusion of phosphatidylcholines in saturated and unsaturated bilayers. Langmuir 2014, 30 (35), 10686–10690. 10.1021/la502338c. 25157681

[ref29] Duda M.; Cygan K.; Wisniewska-Becker A. Effects of curcumin on lipid membranes: An EPR spin-label study. Cell Biochem. Biophys. 2020, 78, 139–147. 10.1007/s12013-020-00906-5. 32236880 PMC7266845

[ref30] Barry J.; Fritz M.; Brender J. R.; Smith P. E.; Lee D.-K.; Ramamoorthy A. Determining the effects of lipophilic drugs on membrane structure by solid-state NMR spectroscopy: the case of the antioxidant curcumin. J. Am. Chem. Soc. 2009, 131 (12), 4490–4498. 10.1021/ja809217u. 19256547 PMC2748423

[ref31] Stillwell W. An Introduction to Biological Membranes: From Bilayers to Rafts; Elsevier Science, 2013. DOI: 10.1016/C2009-0-01475-5.

[ref32] Van Meer G.; Voelker D. R.; Feigenson G. W. Membrane lipids: where they are and how they behave. Nat. Rev. Mol. Cell Biol. 2008, 9 (2), 112–124. 10.1038/nrm2330. 18216768 PMC2642958

[ref33] Funakoshi K.; Suzuki H.; Takeuchi S. Lipid bilayer formation by contacting monolayers in a microfluidic device for membrane protein analysis. Anal. Chem. 2006, 78 (24), 8169–8174. 10.1021/ac0613479. 17165804

[ref34] Holden M. A.; Needham D.; Bayley H. Functional bionetworks from nanoliter water droplets. J. Am. Chem. Soc. 2007, 129 (27), 8650–8655. 10.1021/ja072292a. 17571891

[ref35] Michalak Z.; Muzzio M.; Milianta P. J.; Giacomini R.; Lee S. Effect of monoglyceride structure and cholesterol content on water permeability of the droplet bilayer. Langmuir 2013, 29 (51), 15919–15925. 10.1021/la4040535. 24304231

[ref36] Lee S. Good to the last drop: interfacial droplet chemistry, from crystals to biological membranes. Acc. Chem. Res. 2018, 51 (10), 2524–2534. 10.1021/acs.accounts.8b00277. 30247878

[ref37] Milianta P. J.; Muzzio M.; Denver J.; Cawley G.; Lee S. Water permeability across symmetric and asymmetric droplet interface bilayers: interaction of cholesterol sulfate with DPhPC. Langmuir 2015, 31 (44), 12187–12196. 10.1021/acs.langmuir.5b02748. 26492572

[ref38] Lopez M.; Evangelista S. E.; Morales M.; Lee S. Enthalpic effects of chain length and unsaturation on water permeability across droplet bilayers of homologous monoglycerides. Langmuir 2017, 33 (4), 900–912. 10.1021/acs.langmuir.6b03932. 28073244

[ref39] Lopez M.; Denver J.; Evangelista S. E.; Armetta A.; Di Domizio G.; Lee S. Effects of Acyl Chain Unsaturation on Activation Energy of Water Permeability across Droplet Bilayers of Homologous Monoglycerides: Role of Cholesterol. Langmuir 2018, 34 (5), 2147–2157. 10.1021/acs.langmuir.7b03590. 29323917

[ref40] Foley S.; Miller E.; Braziel S.; Lee S. Molecular organization in mixed SOPC and SDPC model membranes: Water permeability studies of polyunsaturated lipid bilayers. Biochim. Biophys. Acta, Biomembr. 2020, 1862 (9), 183365 10.1016/j.bbamem.2020.183365. 32454009

[ref41] Wood M.; Morales M.; Miller E.; Braziel S.; Giancaspro J.; Scollan P.; Rosario J.; Gayapa A.; Krmic M.; Lee S. Ibuprofen and the Phosphatidylcholine Bilayer: Membrane Water Permeability in the Presence and Absence of Cholesterol. Langmuir 2021, 37 (15), 4468–4480. 10.1021/acs.langmuir.0c03638. 33826350

[ref42] Perez E.; Ceja-Vega J.; Krmic M.; Gamez Hernandez A.; Gudyka J.; Porteus R.; Lee S. Differential interaction of cannabidiol with biomembranes dependent on cholesterol concentration. ACS Chem. Neurosci. 2022, 13 (7), 1046–1054. 10.1021/acschemneuro.2c00040. 35298887

[ref43] Ceja-Vega J.; Perez E.; Scollan P.; Rosario J.; Gamez Hernandez A.; Ivanchenko K.; Gudyka J.; Lee S. Trans-Resveratrol Decreases Membrane Water Permeability: A Study of Cholesterol-Dependent Interactions. J. Membr. Biol. 2022, 255 (4–5), 575–590. 10.1007/s00232-022-00250-0. 35748919

[ref44] Krmic M.; Perez E.; Scollan P.; Ivanchenko K.; Hernandez A. G.; Giancaspro J.; Rosario J.; Ceja-Vega J.; Gudyka J.; Porteus R.; Lee S. Aspirin Interacts with Cholesterol-Containing Membranes in a pH-Dependent Manner. Langmuir 2023, 39 (46), 16444–16456. 10.1021/acs.langmuir.3c02242. 37939382 PMC10666536

[ref45] Yeagle P. L. Cholesterol and the cell membrane. BBA-Rev. Biomembr. 1985, 822 (3–4), 267–287. 10.1016/0304-4157(85)90011-5. 3904832

[ref46] Ohvo-Rekilä H.; Ramstedt B.; Leppimäki P.; Slotte J. P. Cholesterol interactions with phospholipids in membranes. Prog. Lipid Res. 2002, 41 (1), 66–97. 10.1016/s0163-7827(01)00020-0. 11694269

[ref47] Mathai J. C.; Tristram-Nagle S.; Nagle J. F.; Zeidel M. L. Structural determinants of water permeability through the lipid membrane. J. Gen. Physiol. 2008, 131 (1), 69–76. 10.1085/jgp.200709848. 18166626 PMC2174160

[ref48] Olbrich K.; Rawicz W.; Needham D.; Evans E. Water permeability and mechanical strength of polyunsaturated lipid bilayers. Biophys. J. 2000, 79 (1), 321–327. 10.1016/S0006-3495(00)76294-1. 10866958 PMC1300936

[ref49] Stubbs C. D.; Smith A. D. The modification of mammalian membrane polyunsaturated fatty acid composition in relation to membrane fluidity and function. BBA-Rev. Biomembr. 1984, 779 (1), 89–137. 10.1016/0304-4157(84)90005-4. 6229284

[ref50] Sun Y.; Lee C.-C.; Hung W.-C.; Chen F.-Y.; Lee M.-T.; Huang H. W. The bound states of amphipathic drugs in lipid bilayers: study of curcumin. Biophys. J. 2008, 95 (5), 2318–2324. 10.1529/biophysj.108.133736. 18515370 PMC2517021

[ref51] Leite N. B.; Martins D. B.; Fazani V. E.; Vieira M. R.; dos Santos Cabrera M. P. Cholesterol modulates curcumin partitioning and membrane effects. Biochim. Biophys. Acta, Biomembr. 2018, 1860 (11), 2320–2328. 10.1016/j.bbamem.2018.05.018. 29883674

[ref52] Kotenkov S. A.; Gnezdilov O. I.; Khaliullina A. V.; Antzutkin O. N.; Gimatdinov R. S.; Filippov A. V. Effect of cholesterol and curcumin on ordering of DMPC bilayers. Appl. Magn. Reson. 2019, 50, 511–520. 10.1007/s00723-018-1102-2.

[ref53] Koynova R.; Caffrey M. Phases and phase transitions of the phosphatidylcholines. BBA-Rev. Biomembranes 1998, 1376 (1), 91–145. 10.1016/S0304-4157(98)00006-9. 9666088

[ref54] Fritzsching K. J.; Kim J.; Holland G. P. Probing lipid–cholesterol interactions in DOPC/eSM/Chol and DOPC/DPPC/Chol model lipid rafts with DSC and 13C solid-state NMR. Biochim. Biophys. Acta, Biomembr. 2013, 1828 (8), 1889–1898. 10.1016/j.bbamem.2013.03.028. 23567917

[ref55] Lewis R. N.; McElhaney R. N. The structure and organization of phospholipid bilayers as revealed by infrared spectroscopy. Chem. Phys. Lipids 1998, 96 (1–2), 9–21. 10.1016/S0009-3084(98)00077-2.

[ref56] Derenne A.; Claessens T.; Conus C.; Goormaghtigh E. Infrared spectroscopy of membrane lipids. Encycl. Biophys. 2013, 1074–1081. 10.1007/978-3-642-16712-6_558.

[ref57] Blume A.; Hübner W.; Messner G. Fourier transform infrared spectroscopy of 13C: O labeled phospholipids hydrogen bonding to carbonyl groups. Biochemistry 1988, 27 (21), 8239–8249. 10.1021/bi00421a038. 3233207

[ref58] Pohle W.; Selle C.; Fritzsche H.; Binder H. Fourier transform infrared spectroscopy as a probe for the study of the hydration of lipid self-assemblies. I. Methodology and general phenomena. Biospectroscopy 1998, 4 (4), 267–280. 10.1002/(SICI)1520-6343(1998)4:4%3C267::AID-BSPY5%3E3.0.CO;2-%23. 9706385

[ref59] White S. Studies of the physical chemistry of planar bilayer membranes using high-precision measurements of specific capacitance. Ann. N.Y. Acad. Sci. 1977, 303, 243–265. 290294

[ref60] White S. Formation of ″solvent-free″ black lipid bilayer membranes from glyceryl monooleate dispersed in squalene. Biophys. J. 1978, 23 (3), 337–347. 10.1016/S0006-3495(78)85453-8. 698340 PMC1473540

