# Supplementary material for: Concentration-Dependent Effects of Curcumin on Membrane Permeability and Structure
Source: ACS Pharmacol Transl Sci. 2024 Apr 10;7(5):1546–56. doi: 10.1021/acsptsci.4c00093 (PMC11091966; doi:10.1021/acsptsci.4c00093)
Supplement: Supplementary file 1 — pt4c00093_si_001.pdf [file pt4c00093_si_001.pdf]

Supporting Information

Concentration−dependent effects of curcumin on membrane permeability and structure

*Jamie Gudyka, Jasmin Ceja-Vega, Katherine Ivanchenko, Zachary Morocho, Micaela Panella, Alondra Gamez Hernandez, Colleen Clarke, Escarlin Perez, Shakinah Silverberg, and Sunghee Lee**

Department of Chemistry and Biochemistry, Iona University, 715 North Avenue, New Rochelle, New York 10801, USA

*To whom correspondence should be addressed. Tel: 914-633-2638. Fax: 914-633-2240.
E-mail: SLee@iona.edu

1. Analysis of Water Permeability Data

When two osmotically unbalanced microdroplets adhere to a bilayer, the osmotic gradient propels water transport through the droplet bilayer (as indicated by the arrow in Figure 2 in the main article), leading to a noticeable change in droplet diameter. Any electrolyte flux is expected to be negligible compared to that of water, as ion permeation is typically almost eight orders of magnitude slower than that of water. The corresponding changes in droplet volume over time (dV/dt) is measured optically by microscopic observation; and the behavior of the system follows the expression of equation (1) based on Fick's Law:

$$\frac{dV(t)}{dt} = -P_f A(t) \nu_w \Delta C(t) \qquad (1)$$

where A is the geometric bilayer area, ν_w is the molar volume of water (18 mL/mol), **Δ**C(t) is the osmolality gradient between two droplets, and P_f is the bilayer permeability coefficient of water. The volume change with time (dV/dt) is related to the bilayer permeability coefficient of water, P_f, as expressed in the Equation (1). When the bilayer contact area is constant, the time evolution of the swelling droplet can be obtained from the following equation derived from the integration of eqn. 1, with the following simplifying assumption: since one of the droplets (the shrinking droplet) contains no osmotic agent, its concentration does not change with time:[1, 2]

[1] Lopez, M.; Evangelista, S. E.; Morales, M.; Lee, S. Enthalpic effects of chain length and unsaturation on water permeability across droplet bilayers of homologous monoglycerides. *Langmuir* 2017, 33 (4), 900-912.
[2] Thiam, A. R.; Bremond, N.; Bibette, J. From stability to permeability of adhesive emulsion bilayers. *Langmuir* 2012, 28 (15), 6291-6298.

$$\left(\frac{V}{V_o}\right)^2 = \left(\frac{2P_f A v_w C_o}{V_o}\right)t + 1 \qquad (2)$$

Using the measured values for: initial size of the osmotic (swelling) droplet; bilayer contact area (A); and initial osmolarity of the osmotic droplet (C_o), then the coefficient P_f for bilayer water permeability may be derived from eqn. 2 from the slope of the curve obtained by plotting $(V/V_o)^2$ as a function of time. All data points presented in this paper are an average ($n \geq 20$) of individual permeability runs, each of which took place over a time course (~5 min) for osmotic water movement across the droplet bilayer, during which time the droplet contact area (A) remains constant. The recorded videos and images were post-analyzed to measure the dimension of droplets and contact area using custom built image analysis software. All droplet pairs had substantially the same initial size relative to each other, in the diameter range of 100 ± 5 μm diameter.

Table S1. Water permeability coefficient at 30°C for DOPC and DOPC/Chol (4/1 mol/mol) membrane, as a function of curcumin concentratons.

Concentrations of curcumin	Water permeability coefficient (μm/s)	
	DOPC	DOPC/Chol (4/1 mol ratio)
control	74 ± 3	70 ± 3
100 : 1	72 ± 2	69 ± 2
50 : 1	68 ± 2	68 ± 3
30 : 1	74 ± 3	68 ± 2
10 : 1	78 ± 3	67 ± 4
4 : 1	82 ± 6	66 ± 4

2. DSC Thermograms–Deconvolution

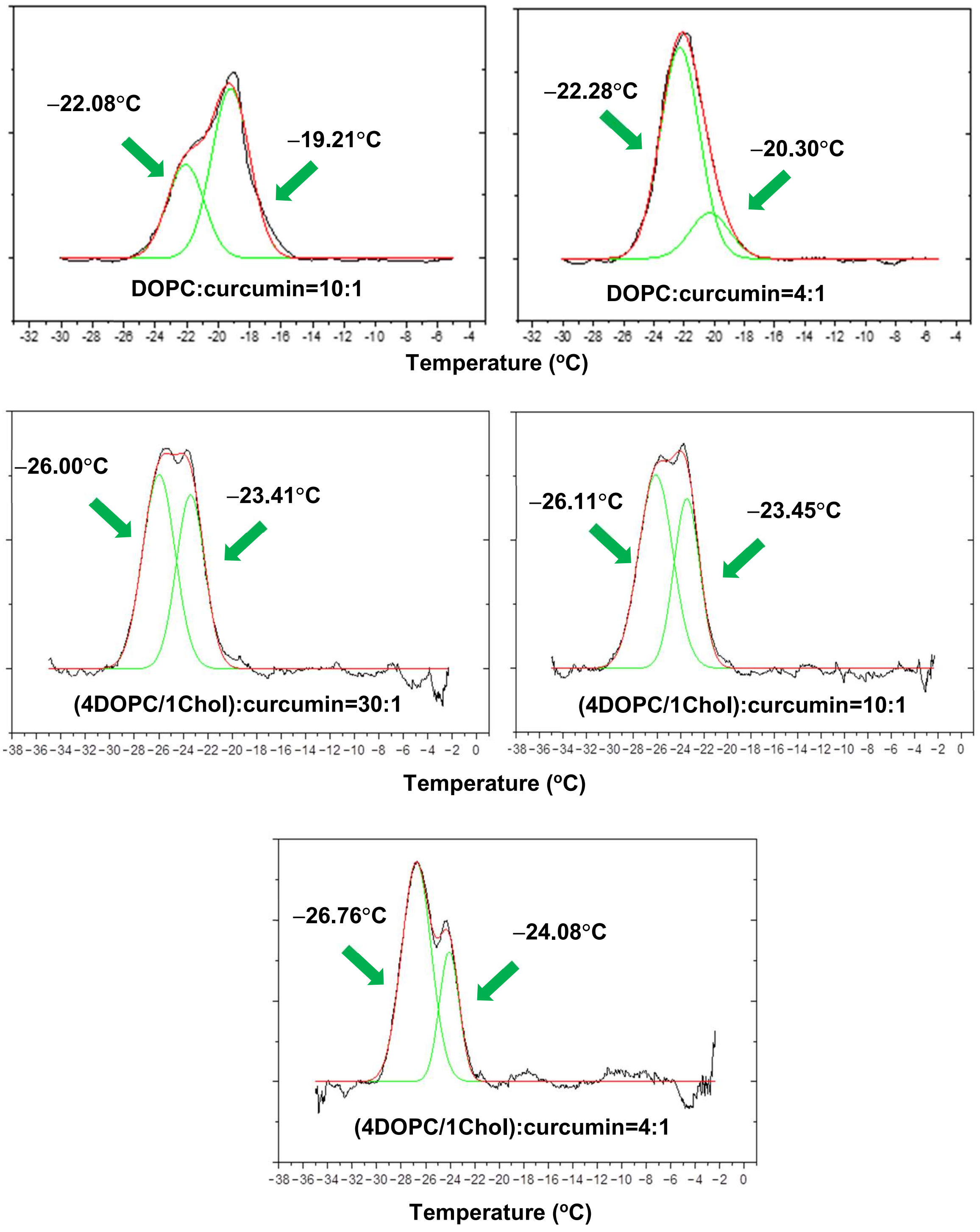

Figure S1. Curve-fitting simulations were performed using OriginPro 9.7 software to deconvolute and fit into two components corresponding to higher T_m (curcumin-poor) and lower T_m (curcumin-rich) regions.

3. ATR-IR Spectra

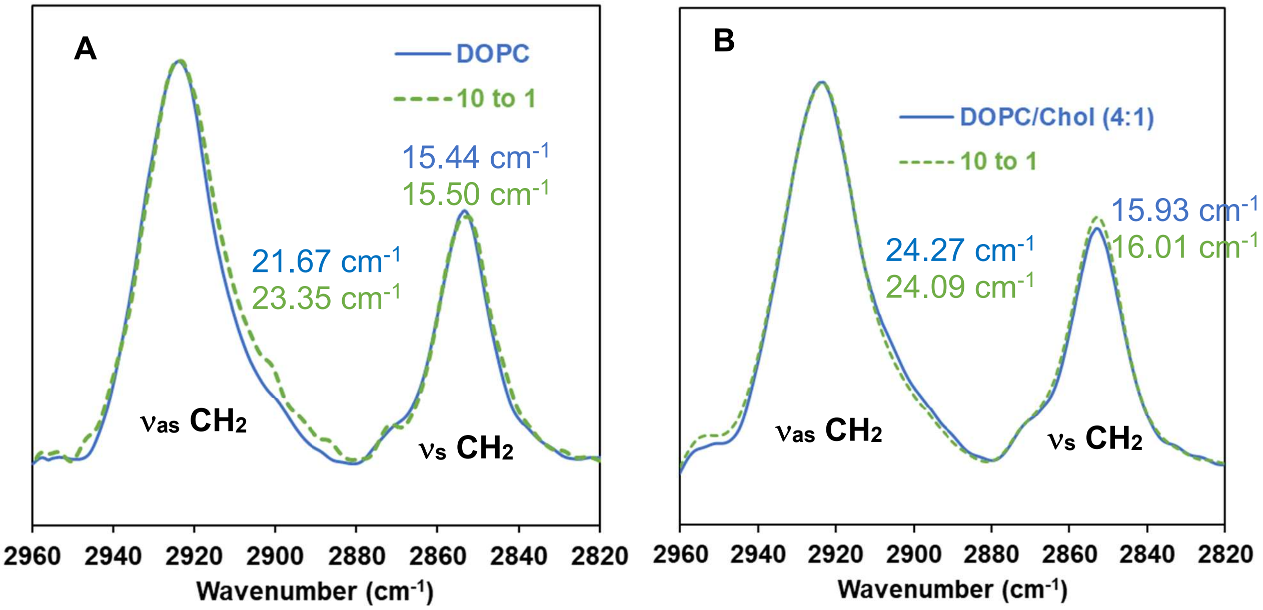

Figure S2. Effect of curcumin on the bandwidth of CH_2 stretching vibration modes in (A) DOPC and (B) DOPC with Chol (4/1 mol ratio) at 10 to 1 mol concentrations of lipid to curcumin. The bandwidth of CH_2 stretching vibration is shown in numbers (blue for DOPC and green for DOPC to Curcumin 10 to 1 mole ratio).

Table S2. Wavenumber with varying concentrations of curcumin at 25°C in the region of antisymmetric PO_2^- stretching vibration bands.

Concentrations of curcumin	Wavenumber (cm^{-1})	
	DOPC	DOPC/Chol (4/1 mol ratio)
control	1229.4 ± 2.4	1227.6 ± 1.9
100 : 1	1227.5 ± 1.7	1225.9 ± 1.5
50 : 1	1222.6 ± 2.7	1223.6 ± 1.3
30 : 1	1222.6 ± 2.7	1225.2 ± 2.3
20 : 1	1228.0 ± 2.0	1226.1 ± 2.0
10 : 1	1230.4 ± 1.4	1227.5 ± 2.6
